# Impact on capsule formation for three different types of implant surface tomography

**DOI:** 10.1038/s41598-022-17320-x

**Published:** 2022-08-08

**Authors:** Hyeon Jun Jeon, MyeongJae Kang, Joon Seok Lee, Jieun Kang, Eun A. Kim, Hee Kyung Jin, Jae-sung Bae, Jung Dug Yang

**Affiliations:** 1 Department of Plastic and Reconstructive Surgery, Kyungpook National University School of Medicine, 130 Dongdeok-ro, Jung-gu, Daegu, 700-421 Korea; 2 Cell and Matrix Research Institute, Kyungpook National University School of Medicine, Daegu, Korea; 3 Exosome Convergence Research Center, Kyungpook National University School of Medicine, Daegu, Korea; 4 Department of Laboratory Animal Medicine, College of Veterinary Medicine, Kyungpook National University, Daegu, Korea; 5 Department of Physiology, Cell and Matrix Research Institute, Kyungpook National University School of Medicine, Daegu, Korea

**Keywords:** Nanoscience and technology, Nanobiotechnology

## Abstract

Although capsular contracture remains one of the major problems following silicone breast implantation, the associated mechanism has yet to be determined. This study thus aimed to investigate capsule formation and capsular contracture using three types of implants with different surface topographies in vivo. Three types of implants (i.e., smooth, macrotexture, and nanotexture) with different surface topographies were inserted in a total of 48 Wistar rats. After 4 and 12 weeks, the samples were analyzed via histological, immunohistochemical, and Western blot examination. To identify implant movement, the degree to which implant position changed was measured. And the surface topography was characterized using scanning electron microscopy. Hematoxylin–eosin staining showed that the nanotexture type implant promoted significant decreases in capsule thickness at 12 weeks (*P* < 0.05), while Masson trichrome staining showed decreased collagen fiber density with the same implant type. Immunohistochemical and Western blot examination revealed reduced fibrosis markers (myofibroblast, and transforming growth factor beta-1) in the nanotexture surface implant. Meanwhile, implant location evaluation found that the nanotexture and smooth surface implants had significantly increased movement (*P* < 0.05). The nanotexture surface implant had been found to reduce capsule formation given that it minimizes the effects of factors related to foreign body reaction.

## Introduction

Silicon remains one of the most widely used medical materials, with breast silicone implants having been used ubiquitously until recently in the field of plastic surgery for breast augmentation and breast reconstruction since their introduction in 1963 by Cronin and Gerow^1,2^. However, complications, such as capsular contracture, malposition, rupture, and skin rippling have been reported, among which capsular contractures exhibit the highest incidence rates (15–30%)^3,4^. Although various theories regarding their occurrence have been posited, such as foreign body reaction, anatomical location of the implant, asymptomatic infection or inflammation, bacterial biocapsule, and material property of the implant surfaces, the exact etiology and mechanism of capsular contraction have yet to be clearly established^5,6^.

Throughout their history, surface implants have shifted from the previous smooth type to the current textured type to supplement their shortcomings, which range from malposition to capsular contracture. Notably, several animals and clinical studies have observed differences in occurrence rate of capsular contraction depending on the surface implants used^7–9^. Accordingly, capsular contracture has been associated with the surface characteristics of implants, with surface topography, in particular, affecting attachment, proliferation, migration, differentiation of various types of cells, and connective tissues^10–13^. In addition, interest in surface implants has increased recently because of the occurrence of new side effects such as not only late seroma but also breast implant associated-anaplastic large cell lymphoma due to aggressive texturization of the rough surface of implants^14,15^.

Based on the hypothesis that the surface implant is related to foreign body reaction, film formation as well as capsular contracture, many studies are being conducted on surface modification with respect to the physiological and chemical aspects in order to solve this issue. For instance, topography modification through difference in pore size or roughness, surface polarization modification through oxygen plasma activation, and surface modification using a coating of anti-fibrosis drugs, such as triamcinolone or montelukast, among others^16–18^. Although the nanotexture type implant was developed and used recently after the development of smooth surface and macrotexture surface, additional studies are still required to definitively determine the etiology and management of capsular contractures.

In relation on capsular contracture, this study conducted an animal experiment using three different types of implants, namely a recently developed nanotexture surface implant; the existing smooth surface implant; and a macrotexture surface implant, after which capsule formation and their effects were compared and analyzed.

## Results

### Implant surface topography

**Figure 1:**
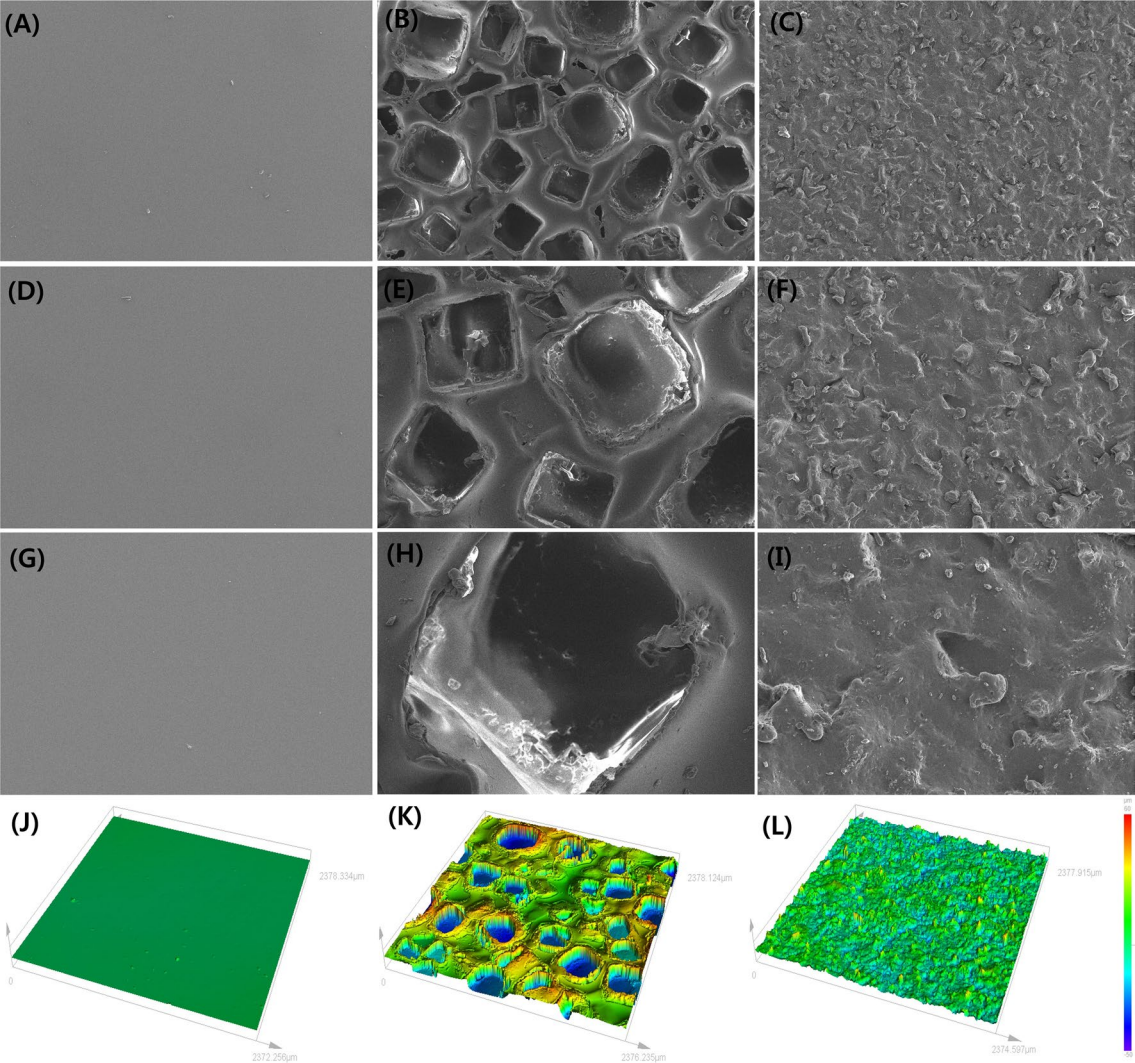
Implant surface topography measured by scanning electron microscopy and 3D confocal laser scanning microscopy. Scanning electron microscopy images of the three types of implants with different topographies. (**A**) smooth type (top view × 50) (**B**) macrotexture type (top view × 50), (**C**) nanotexture type (top view × 50), (**D**) smooth type (top view × 100), (**E**) macrotexture type (top view × 100), (**F**) nanotexture type (top view × 100), (**G**) smooth type (top view × 300), (**H**) macrotexture type (top view × 300), (**I**) nanotexture type (top view × 300). 3D confocal laser scanning microscope images of three types of implants with different topographies. The roughness was measured to be 0.53 ± 0.14, 104.82 ± 7.49, and 6.53 ± 0.25 µm in the smooth, macrotexture, and nanotexture type implants, respectively: (**J**) smooth type, (**K**) macrotexture type and (**L**) nanotexture type.

The smooth surface implant showed relative uniformity when observed through the scanning electron micrograph, and the surface was shown to be smooth almost without any structural texturing such as peak, valley, etc. The rough surface implant showed irregularly arranged cube-like cavities of different sizes. In contrast to smooth surface implant, the nano-texture surface implant was seen to have random and rugged surface. When compared with rough surface implant, it had a relatively regular and uniform shape. The roughness was identified through a three-dimensional (3D) confocal laser scanning microscope. The roughness of the smooth surface implant was 0.40 ± 0.20 µm, 100.10 ± 10.40 µm in rough surface implant, and 5.96 ± 0.41 µm in nano-texture surface implant (Fig. 1).

### Capsule thickness

**Figure 2:**
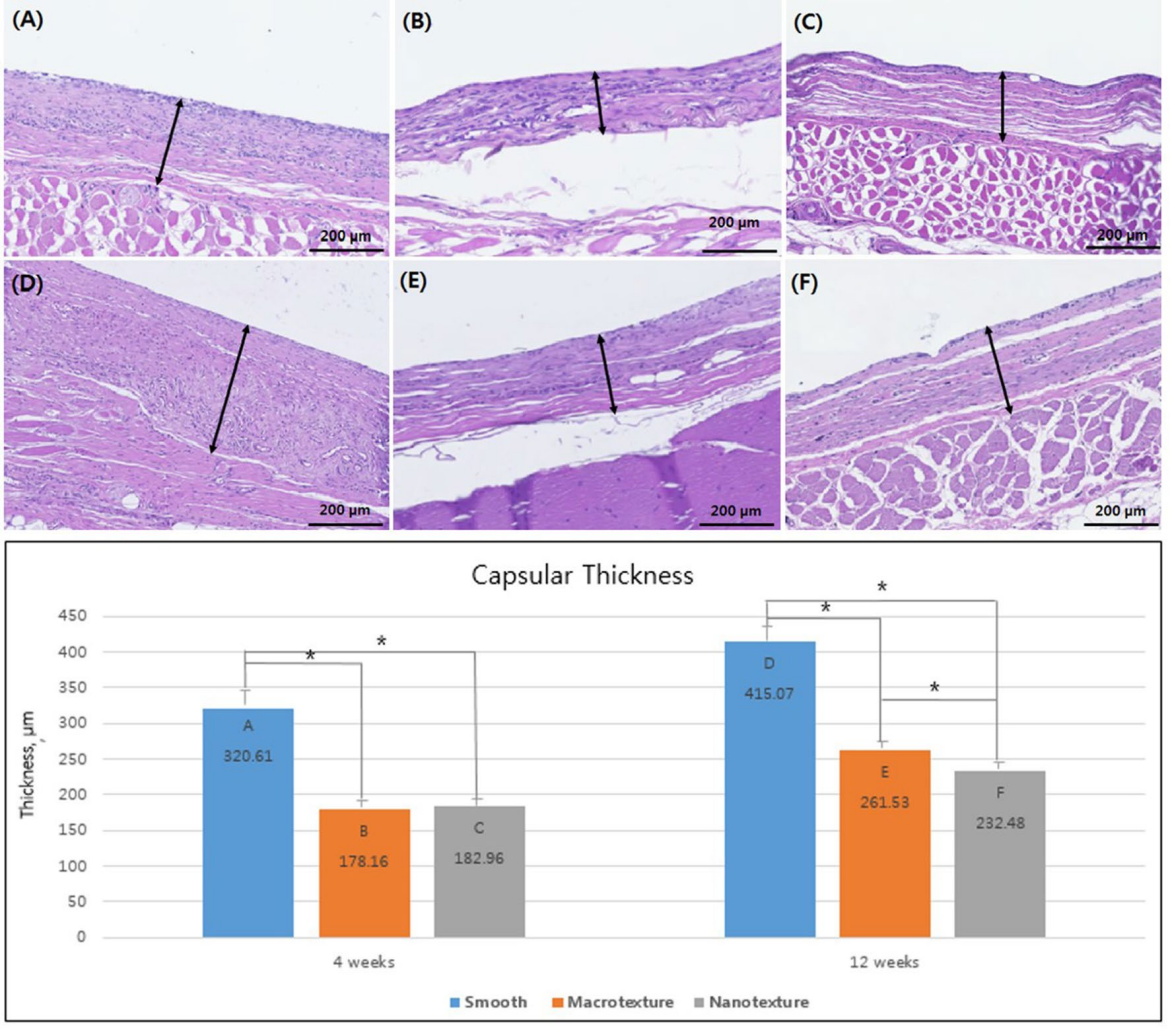
Capsule formation of the three different types of silicone implants. Representative histologic sections of capsular tissues on the silicone implants showing capsular thickness (original magnification, × 100; scale bar = 200 μm). Sections were stained with hematoxylin and eosin. The bar graph shows the thickness of capsule. The black asterisks in the bars represent *P* values indicating significant differences between the groups (*P* < 0.05). (**A**) Group A (4 weeks, smooth type), (**B**) Group B (4 weeks, macrotexture type), (**C**) Group C (4 weeks, nanotexture type), (**D**) Group D (12 weeks, smooth type), (**E**) Group E (12 weeks, macrotexture type), (**F**) Group F (12 weeks, nanotexture type).

H&E staining related a capsule thickness of 320.61 ± 25.75, 178.16 ± 4.10, 182.96 ± 4.83, 415.07 ± 19.74, 261.53 ± 5.7, and 232.48 ± 14.10 µm in groups A, B, C, D, E, and F, respectively. Four weeks after the surgery, groups A (smooth) showed the greatest increase in capsule thickness, followed by groups C (nanotexture) and B (macrotexture). Significant differences in thickness between groups A and B and between groups A and C were observed, with no significance difference having been noted between groups B and C. Twelve weeks after the surgery, groups D (smooth) showed the greatest increase in capsule thickness, followed by groups E (macrotexture), and F (nanotexture), with our results confirming a significant difference in thickness between each group (*P* < 0.05) (Fig. 2).

### Density and arrangement of collagen fiber

**Figure 3:**
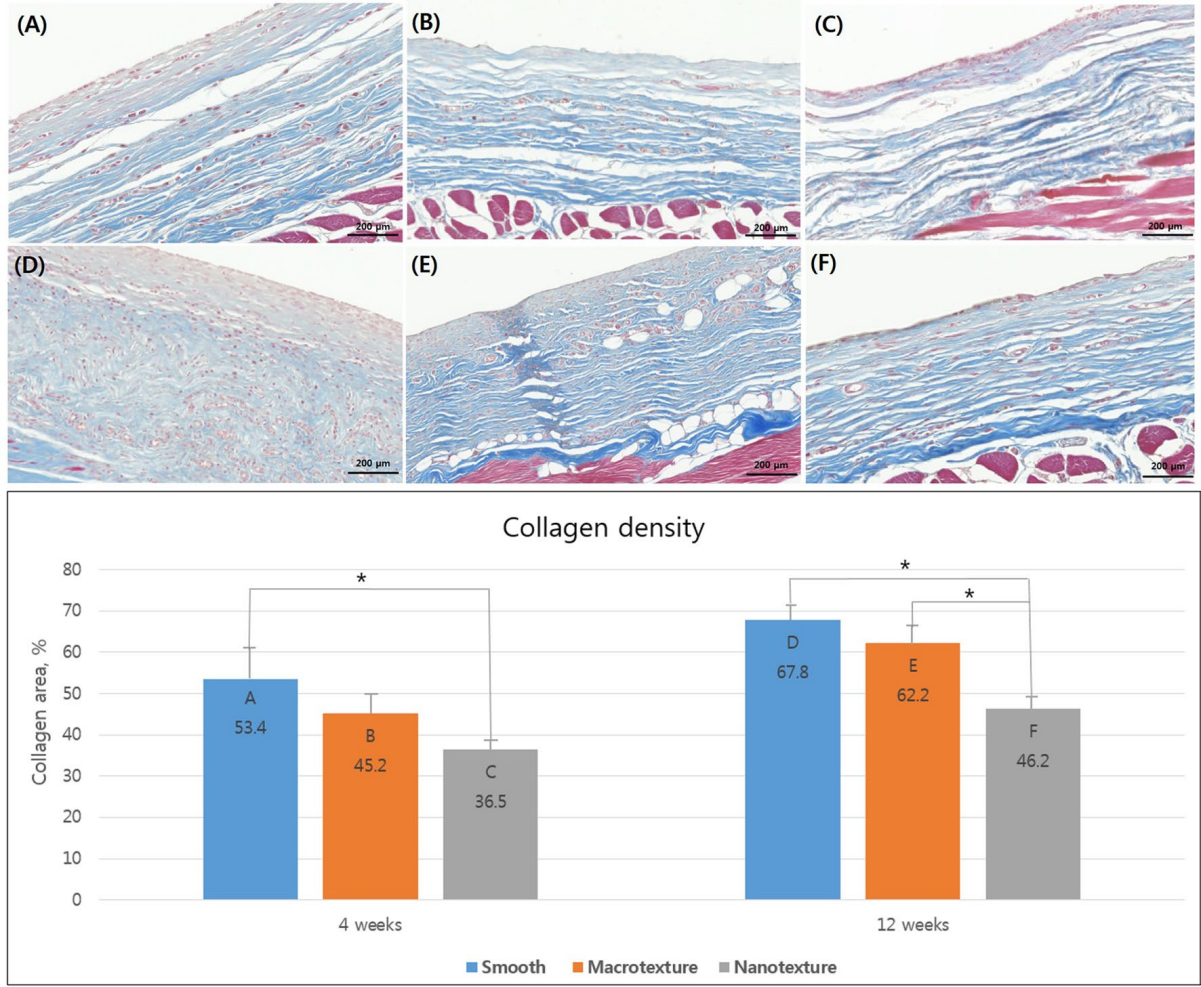
Collagen density and arrangement across the three different types of silicone implants. Representative histologic sections of capsular tissues on the silicone implants showing collagen density and arrangement. Sections were stained with Masson trichrome (original magnification × 100; scale bar = 200 μm). Collagen density measurement according to collagen area. The black asterisks in the bars represent *P* values indicating significant differences between the groups (*P* < 0.05). (**A**) Group A (4 weeks, smooth type), (**B**) Group B (4 weeks, macrotexture type), (**C**) Group C (4 weeks, nanotexture type), (**D**) Group D (12 weeks, smooth type), (**E**) Group E (12 weeks, macrotexture type), (**F**) Group F (12 weeks, nanotexture type).

Masson trichrome staining showed that groups A (smooth), B (macrotexture), and C (nanotexture) had a collagen density of 53.4 ± 8.4%, 45.2 ± 5.7%, and 36.5 ± 2.9% 4 weeks after the surgery, respectively, with group A (smooth) having the greatest increase in collagen fiber density and elaborateness of arrangement, followed by groups B (macrotexture) and C (nanotexture). Moreover, our results confirmed that the nanotexture surface implant had a significantly greater decrease in density compared to the smooth surface implant. Twelve weeks after the surgery, the collagen fiber density formed in the capsule of groups D (smooth), E (macrotexture), and F (nanotexture) was 67.8 ± 3.4%, 62.2 ± 6.1%, and 46.2 ± 3.3%, with the decrease in the density and elaborateness of the arrangement following the same order. Moreover, the nanotexture surface implant had a significantly greater decrease in density compared to the smooth surface and macrotexture surface implants (Fig. 3).

### Immunohistochemical examination

**Figure 4:**
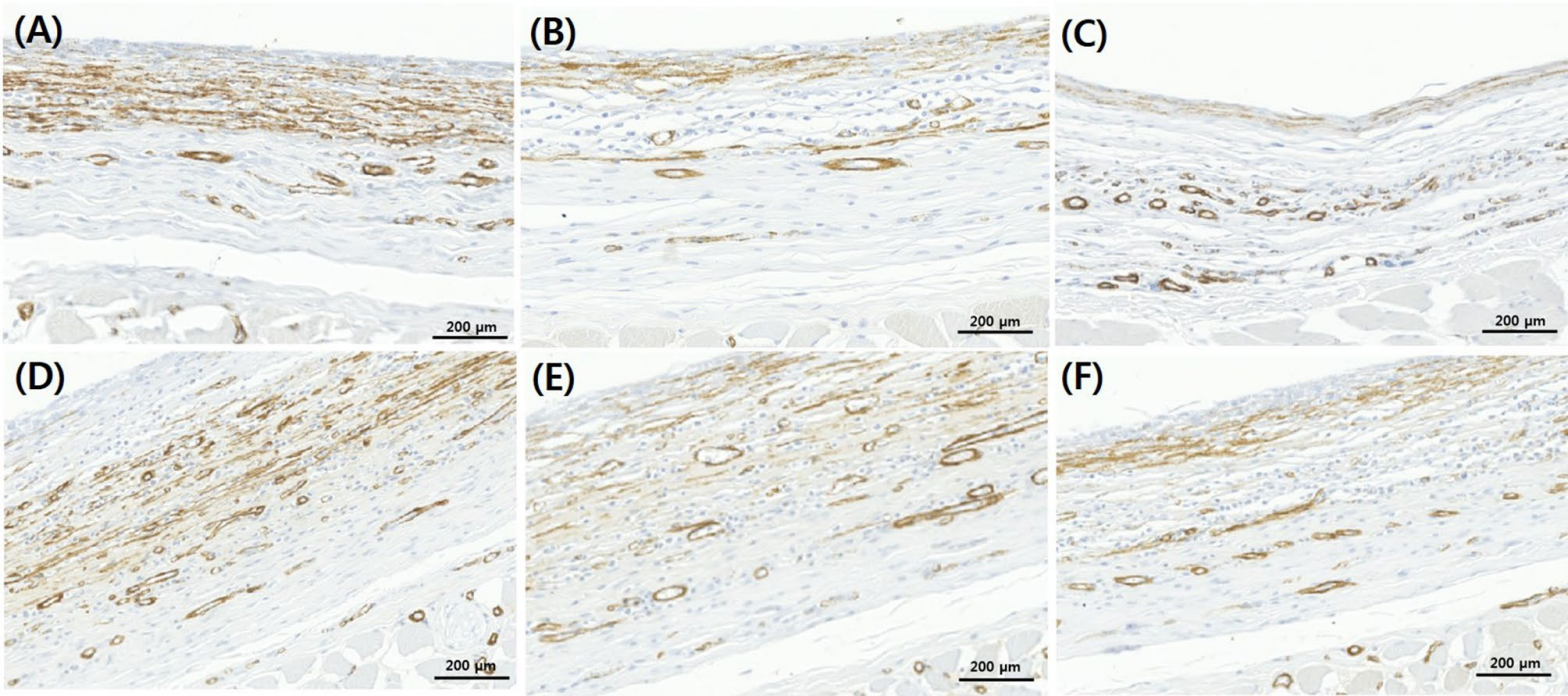
Immunohistochemical examination of myofibroblasts in the three different types of silicone implants. Representative immunohistochemistry sections of capsular tissues on the silicone implant (original magnification × 200). Sections were stained with anti-smooth muscle antibody antibody. (**A**) Group A (4 weeks, smooth type), (**B**) Group B (4 weeks, macrotexture type), (**C**) Group C (4 weeks, nanotexture type), (**D**) Group D (12 weeks, smooth type), (**E**) Group E (12 weeks, macrotexture type), (**F**) Group F (12 weeks, nanotexture type).

Immunohistochemical examination showed that groups A (smooth), B (macrotexture), and C (nanotexture) had a myofibroblast infiltration of 31.4 ± 8.4%, 14.2 ± 3.7%, and 10.5 ± 2.9% 4 weeks after the surgery, respectively. Twelve weeks after the surgery, the myofibroblast infiltration of groups D (smooth), E (macrotexture), and F (nanotexture) was 42.8 ± 3.4%, 25.2 ± 6.1%, and 16.2 ± 3.3%. There was no statistical significance, but the nanotexture surface implant showed a relatively decreased result in myofibroblast infiltration than the smooth surface and macrotexture surface implants. (Fig. 4).

### Western blot examination

**Figure 5:**
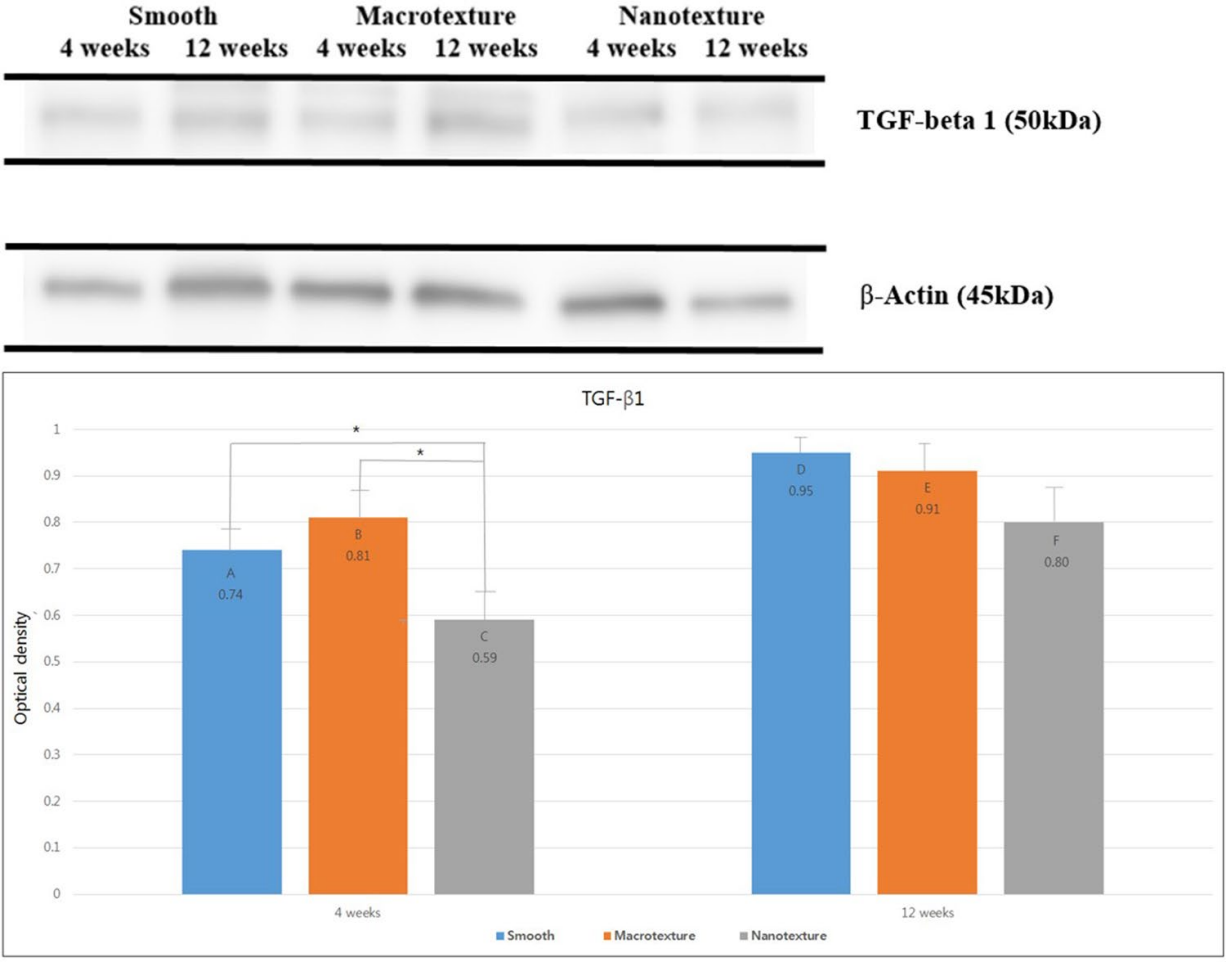
Western blot analysis of TGF-β1 in the three different types of silicone implants. Expression of TGF-β1 and β-Actin were analyzed using Western blotting. Bar graph showing the optical density of TGF-β1. Black asterisks in the bars represent *P* values indicating significant differences between the groups (*P* < 0.05). Group A (4 weeks, smooth type), Group B (4 weeks, macrotexture type), Group C (4 weeks, nanotexture type), Group D (12 weeks, smooth type), Group E (12 weeks, macrotexture type), Group F (12 weeks, nanotexture type). Additional information is mentioned in supplementary information.

After quantitatively comparing the degree of TGF-β1 expression, our results showed an optical density of 0.74 ± 0.05, 0.81 ± 0.08, and 0.59 ± 0.05 in groups A (smooth), B (macrotexture), and C (nanotexture) 4 weeks after the surgery. The nanotexture surface implants showed significantly greater decrease in expression compared to the smooth and macrotexture surface implants (*P* < 0.05). The optical density 12 weeks after the surgery was 0.95 ± 0.04, 0.91 ± 0.05, and 0.80 ± 0.09 in groups D (smooth), E (macrotexture), and F (nanotexture), with group D (smooth) showing the greatest decrease in optical density, followed by groups E (macrotexture) and F (nanotexture), although no significant difference was found between the groups (Fig. 5).

### Silicone implant location

The location of each implant measured at 1, 2, 4, and 12 weeks after implantation was at 90.50° ± 62.67°, 51.00° ± 46.40°, 114.50° ± 6.03°, and 80.75° ± 50.60° for the smooth surface implant; 116.50° ± 18.70°, 127.50° ± 24.37°, 134.25° ± 30.06°, and 92.25° ± 56.94°, for the macrotexture surface implant; and 76.05° ± 38.19°, 115.25° ± 38.53°, 58.75° ± 44.77°, and 29.75° ± 54.86° for nanotexture surface implant, respectively.

**Figure 6:**
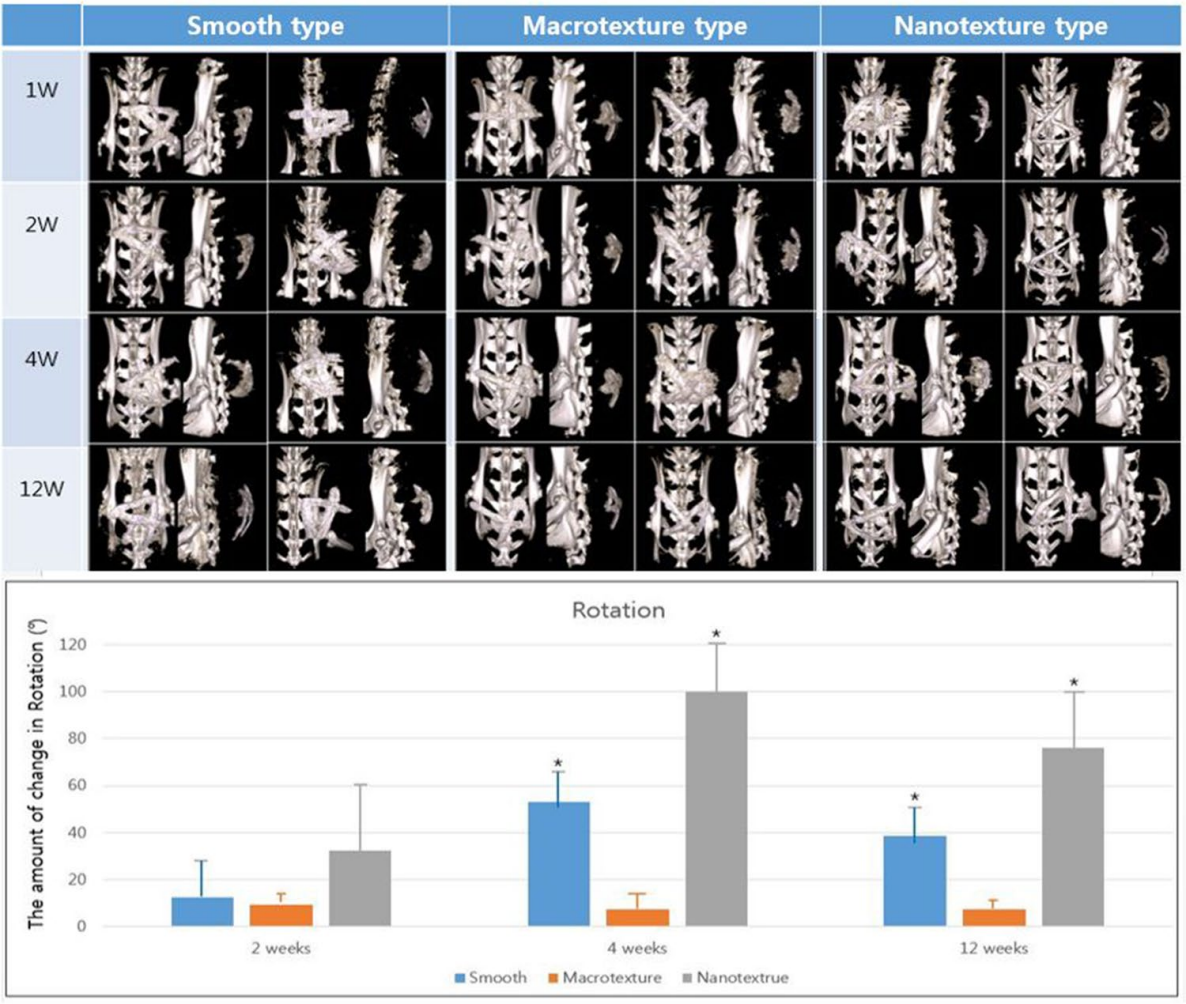
Location of the three different types of silicone implants. Micro-computed tomography scans performed at 1, 2, 4, and 12 weeks after breast implant insertion. Three types of implants with different topographies (smooth, macrotexture, and nanotexture type). Bar graph showing the mean value of rotation. The amount of change in rotation was measured at 2, 4, and 12 weeks from the previous position. Black asterisks in the bars represent *P* values indicating significant differences between the groups (*P* < 0.05).

Moreover, the change in location of each implant from the previous location at 2, 4, and 12 weeks was 16.40° ± 17.67°, 53.00° ± 15.53°, and 38.75° ± 15.56° for smooth surface implant; 12.0° ± 3.06°, 7.33° ± 7.77°, and 3.50° ± 1.73° for the macrotexture surface implant; and 32.50° ± 28.55°, 100.00° ± 21.52°, and 76.00 ± 24.01° for nanotexture surface implant, respectively. These results showed that the magnitude at which the location changed was greatest in the nanotexture surface implant, followed by the smooth and macrotexture surface implants. Our results confirmed that the nanotexture and smooth surface implants had a significantly greater change in location compared to the macrotexture surface implant (*P* < 0.05), although no significant difference was found between the nanotexture and smooth surface implants (Fig. 6).

## Discussion

From the first to the current fifth generation, silicone breast implants have undergone various developmental advancements by addressing potential shortcomings based on surface and gel component attributes^14,15,19,20^. However, the etiology and mechanism of capsular contracture, the most problematic complication, have yet to be clearly known established. On the other hands, some studies have been conducted based on foreign body reaction, which has been considered the most reliable cause for complications^2,6,9^.

Recently, nanotexture surface implants have continued to be developed and used. In ISO 2018, any surface with roughness below 10 is classified as smooth, 10–50 micro texture and > 50 macro texture. Especially, in a paper published by Barr S. et al., a surface with a roughness smaller than 5 μm was classified as nanotexture^21^. Among the implants we used in our experiment, the roughness value of the nanotexture surface was about 6 μm, and as other papers mentioning nanotexture show a value of about 3–5 μm, the standard is not clear. However, the term nanotexture is gradually being used as a number of studies have been conducted recently. In order to distinguish it from the existing smooth, microtexture, and macrotexture, I would like you to understand the definition of nanotexture in consideration of the relative meaning of nano. Unlike past implants with smooth or macrotexture surface, nanotexture surface implants have a relatively biomimetic feature from the topographical perspective. Thus, such implants have been considered to have higher biocompatibility given that it creates a familiar biological microenvironment with surrounding cells by affecting the cell signal transduction mechanism through natural interaction between the implant and surrounding cells, as well as with cytokine release^22,23^.

Several studies have already shown that although no significant difference in the occurrence rate of contracture had been observed between smooth and macrotexture surface implants, macrotexture surface implants promote thinner capsules and have lower density and less dense alignment of collagen fibers near the implant compared to smooth surface implants. Moreover, evidence has shown that macrotexture surface implants facilitate a decrease in the number and expression of myofibroblasts and transforming growth factor beta-1^7,12^. The current study also showed tendencies similar to that presented in the aforementioned study. In addition, our results showed that nanostructure surface implants promoted a lower capsule thickness, collagen fiber density and arrangement, and the expression of myofibroblasts and transforming growth factor beta-1 compared to both the smooth and macrotexture surface implants, with such decreases these clearly occurring over time. Our experimental analysis therefore suggests that nanotexture surface implants may be an effective approach toward reducing capsular contracture given that it addresses the adverse effects to surrounding tissues caused by foreign body reactions to the implant. Similarly, a recent clinical study showed that nanotexture surface implants, categorized as nano-textured type, was consequently safe and had lesser side effects, including double capsule, implant damage, capsular contracture, and seroma^24^. Moreover, evidence has shown that the close interaction between the implant surface and cells surrounding the capsular contracture reduces macrophage activity, which reduces the occurrence of complications and supports the compatibility of nanotexture surface implant^24,25^.

After investigating the degree to which the implant location changed by rotation, our findings confirmed that nanotexture surface implants exhibited the greatest change in location, followed by smooth surface, and macrotexture surface implants. One reason why the nanotexture surface implant changed locations the most may be its thinner formed capsule compared to the smooth and macrotexture surface, as observed visually. Our results confirmed that the macrotexture surface implant changed locations the least given its firm attachment owing to its high fractional force with its surrounding tissues. Therefore, using nanotexture surface implants in clinical practice can reduce side effects, such as capsular contracture, given its thinner capsule formed upon implantation. However, this may lead to considerable changes change in implant location. Based on clinical experience and other literatures, excessive dissection in implant pocket increases the chance of implant malposition, such as bottoming-out or double bubble, when creating a space for the implant. Therefore, it is recommended to make the dissection of a very tight pocket to minimize inferior and lateral migration during surgery^26^. And also, specific surgical technique, composite reverse inferior muscle sling (CRIMS), helps the surgeon accurately place the silicone gel implant with more stability^27,28^.

Furthermore, the current study utilized a 3D confocal laser scanning microscope to measure the roughness, assumed to be a frictional force between the implant surface and its surrounding tissues, across the three different implant types. Accordingly, the macrotexture surface implant developed more cracks in the surrounding tissues give that roughness is proportional to frictional force, which can promote persistent inflammatory reactions. However, smooth surface implants are known to minimize the inflammatory reaction considering that it exerts less frictional force onto its surrounding surface^29^. Nonetheless, considering that that capsular contracture occurred more frequently in the smooth surface than in the macrotexture surface implant, frictional force inferred by the roughness and other causes, such as the interaction between the implant and its surrounding tissue, including tissue ingrowth, need to be considered. Unlike macrotexture surface implants where cells are randomly arranged based on the difference of surface morphology, smooth surface implant have cells that are arranged more uniformly, which promotes excessive fibroblast growth that eventually develops into a capsular contracture. The behavior of fibroblasts can be identified through their filopodia, with studies showing that cell migration and proliferation are worse in smooth surface compared to macrotexture surface implants, which may have an impact on capsular contracture^30^. This shows that frictional force in nanotexture surface implants is insufficient to cause cracks in surrounding tissues while having topography similar to normal surrounding cells, unlike smooth surface implants. This may reduce inflammatory and foreign body reactions, as well as capsular contractures.

The study limitations include the lack of correlation analysis between skewness, kurtosis, and contract angle, which, apart from roughness, have been associated with surface topography. Next, if it was possible to confirm the additional experimental design and results for macrophage or fibroblast, which are factors that play an important role in each step of the foreign body reaction, the effect of each implant on the capsular contracture in terms of surface topography will be further demonstrated. In addition, due to low costs, availability and easy handling, murines are popular experimental models to study performance of biomaterials in vivo. However, murine healing and response to biomaterials does not exactly reflect response to biomaterials in human or in larger animals. In human or larger animals, inflammatory reactions and foreign body reactions could be even worse. If we apply this to larger animals or humans based on the research process and results in the future, It would have been able to help with the future direction in capsular contracture research.

## Materials and methods

The animal experiment was conducted after review and obtaining approval from the Institutional Animal Care and Use Committee of Daegu-Gyeongbuk Medical Innovation Foundation (DGMIF) (approval number: DGMIF-19073103-01). This study was carried out in compliance with the ARRIVE guidelines and all experiments were carried out in strict accordance with the recommendations in the Guide for the Care and Use of Laboratory Animals of the National Institutes of Health.

### Breast implants

**Figure 7:**
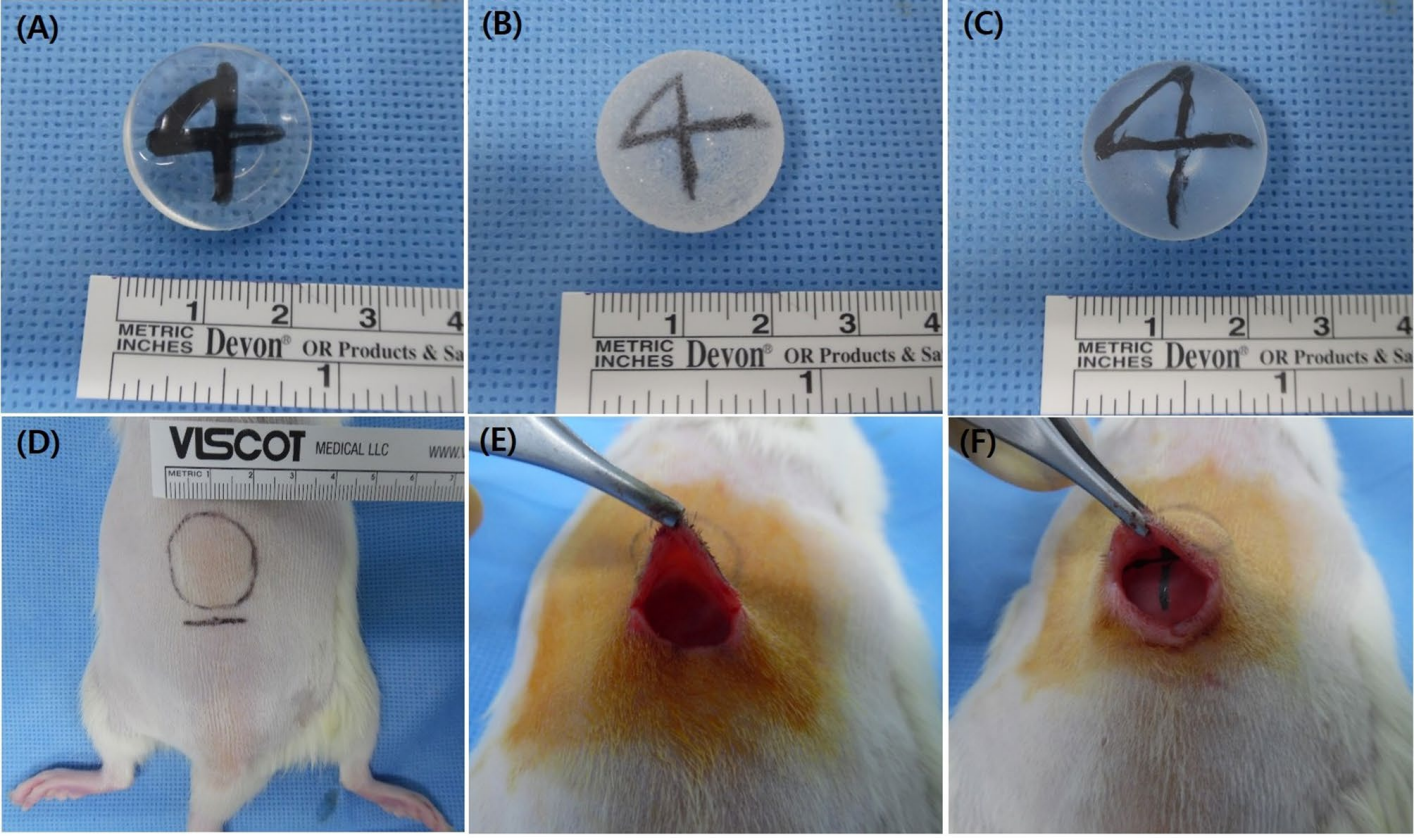
Three types of implants and surgical implantation procedure. (**A**) Smooth, (**B**) macrotexture, and (**C**) nanotexture types: Three types of implants with different topographies that have a directional marker made of tantalum specially manufactured by Hans Daeduk Engineering Research Lab (Daejeon, Korea), a hemispherical shape, a diameter of 2 cm, and a volume of 2 cm^3^. (**D**) The design was made on the back area of Wister rats, while the incision line was approximately 2 cm at the place where the implant is expected to be located. (**E**) Dissection with metzenbaum was done under the panniculus carnosus muscle. (**F**) The implant was placed under the panniculus carnosus muscle.

The experiment was conducted using a specially manufactured hemispherical implant (Hansbiomed Co., Ltd., Seoul, Korea) with three different types of topographies (smooth, macrotexture, and nanotexture surface) with a diameter of 2 cm and volume of 2 cm^3^. The surface of three different implants is composed of polydimethylsiloxane and does not contain any other material. Moreover, a tantalum marker with the ability to determine location was inserted within the implants to detect their location (Fig. 7).

### Measurement of surface topography

The surface of each implant was confirmed using an electron microscope (scanning electron micrographs, S-4200, Hitachi, Tokyo, Japan). Shell samples having a diameter of 1.5 cm were acquired from each of three different implant types (smooth surface, rough surface, and nano-texture surface). The acquired samples were coated with Pt after washing with isopropyl alcohol and then dried. The surfaces measured at 50, 100, and 300 times in 10.0 kV, and 100 times with 20° tilted angle, were analyzed.

Moreover, the roughness of the surface implant was measured using a 3D confocal laser scanning microscope (LEXT OL5000, Olympus Corporation, Tokyo, Japan). Based on the surface topography (smooth surface, rough surface, and nano-texture surface), 2 cm^2^-sized shell samples were acquired from each of the three different implant types, which were then washed with isopropyl alcohol and dried. Following this, a 2 mm × 2 mm area of the samples was measured using 20 × lenses, and analysis was conducted on the roughness of the measured surface.

### Animal experiment

A total of 48 10-week-old Wistar rats (Orientbio, Seongnam, Gyeonggido, Korea) weighing from 200 to 250 g were each subjected to general anesthesia via intraperitoneal administration of a mixture of 10 mg xylazinehydrochloride/kg (Rompun®; BayerKorea, Korea) and 30 mg of tiletamine/zolazepam/kg (Zoletil®50; Virbac, Korea). A 2-cm diameter incision was made in the back through which a hemispheric silicone implant was inserted under the panniculus carnosus.

Rates were classified into a total of six experimental groups: group A wherein the smooth surface implant was used and capsule extraction was made 4 weeks after implantation; group D at 12 weeks after; groups B and E wherein the macrotexture surface implant was used and capsule extraction was made 4 and 12 weeks after implantation, respectively; and groups C and F wherein the nanotexture surface implant was used and capsule extraction was made at 4 and 12 weeks after implantation, respectively. Moreover, micro-computed tomography (CT) (Quantum FX) was performed 1, 2, 4, and 12 weeks after implantation to determine changes in embedded implant location over time. This animal experiment was approved by the Institutional Animal Care and Use Committee of Daegu-Gyeongbuk Medical Innovation Foundation (approval no. DGMIF-19073103-01) and was conducted in accordance with the committee’s recommendations.

### Histological examination

Paraffin blocks were made within 24 h after the capsule tissues extracted along with implants were fixed in 10% paraformaldehyde. Tissue sections with a thickness of 3 μm were made from all samples. Thereafter, hematoxylin and eosin (H&E) staining and Masson trichrome staining were performed. For analysis, digital images of the tissue specimens were created using a slide scanner (Axio Scan. Z1; Carl Zeiss A/S, Birkeroed, Denmark). To measure the thickness of the capsules during H&E staining, a 1200 × 800 μm-sized area was defined in each digital image using Zeiss Zen software Ver. 2 (Carl Zeiss GmbH, Jena, Germany). Subsequently, All analysed used 25 stined slides for each group and the average thickness of six parts at 200-μm intervals was calculated for each capsule. Furthermore, the density and arrangement of collagen fibers within the capsules in Masson trichrome staining were confirmed using Zeiss Zen software Ver. 2. After selecting five different parts within the same image, collagen-stained areas were measured using the ImageJ program (1.43 u; http://rsbweb.nih.gov/ij/). Lastly, a comparison analysis was conducted using the aforementioned data.

### Immunohistochemical examination

To analyze myofibroblasts, which are fibrosis-related factors, anti-smooth muscle antibody (SMA) (ab12512; Abcam, Cambridge, MA, USA.) was used as the primary antibodies and DAKO K4001 was used as the secondary antibodies. The extracted capsule tissues were fixed in 10% paraformaldehyde, immersed in 95% ethanol for 5 min, and then fixed by washing in distilled water. They were processed in hydrogen peroxide for 5 min, washed with Tris-buffered saline (TBS), cultured after the addition of the primary antibody, and finally washed with TBS. The secondary antibody was then added, after which tissue samples were cultured. They were then analyzed after washing with distilled water and counter staining with hematoxylin following staining with substrate-chromogen solution.

For specimen analysis of the anti-SMA antibody, digital imaging was performed on tissue specimen using a slide scanner (Axio Scan. Z1; Carl Zeiss A/S, Birkeroed, Denmark), after which the degree of each expression was identified using Zeiss Zen software Ver. 2. and ImageJ program (1.43 u; http://rsbweb.nih.gov/ij/).

### Western blot examination

Proteins were extracted from formalin-fixed paraffin-embedded (FFPE) tissue using the Qproteome FFPE Tissue Kit (QIAGEN, Mettmann, Germany) according to the manufacturers’ instructions. Extracted proteins were separated using sodium dodecyl sulfate–polyacrylamide gel electrophoresis and blotted on a polyvinylidene difluoride membrane. Membranes were blocked with 5% skim milk powder in Tris-buffered saline [20 mM Tris–HCl and 137 mM NaCl (pH 7.6)] containing 0.1% Tween-20 (TBS-T buffer) for 2 h at room temperature. Membranes were then incubated with antibodies against TGF-beta 1 (Abcam, Cambridge, UK) or β-actin (Cell Signaling Technology, Danvers, MA, USA) overnight at 4 °C. The samples were washed thrice with TBS-T buffer and incubated with the secondary antibodies (Cell Signaling Technology, Danvers, MA, USA) for 1 h at room temperature. After the membranes were washed thrice, protein bands were detected using a chemiluminescence reagent (Thermo Scientific, Waltham, MA, USA). Bands were quantified using ImageJ program (1.43 u; http://rsbweb.nih.gov/ij/).

### Location measurement

To confirm changes in embedded implant location over time, micro-CT (Quantum FX) was performed 1, 2, 4, and 12 weeks after implantation. To determine changes in location, the degree of rotation for each implant was measured. For this purpose, the point of the compass within the implant was marked using tantalum, while the rotation angle was measured clock-wise or counter-clock-wise based on the marked points on the compasses upon implantation. A degree of rotation no more than 180° was selected.

### Statistical analysis

Measured values were statistically analyzed using SPSS 23.0 (IBM, Armonk, NY, USA) and presented as average ± standard deviation. Two-way ANOVA and Tukey’s multiple comparison post-hoc tests were used to determine significant differences between the experimental groups, with *P* < 0.05 indicating statistical significance.

### Ethics approval for animal experiments

The animal experiment was conducted after review and obtaining approval from the Institutional Animal Care and Use Committee of Daegu-Gyeongbuk Medical Innovation Foundation (DGMIF) (approval number: DGMIF-19073103-01). This study was carried out in compliance with the ARRIVE guidelines and all experiments were carried out in strict accordance with the recommendations in the Guide for the Care and Use of Laboratory Animals of the National Institutes of Health.

## Supplementary Information

Supplementary Information.
