# Supplementary material for: Impact on capsule formation for three different types of implant surface tomography
Source: Sci Rep. 2022 Aug 8;12:13535. doi: 10.1038/s41598-022-17320-x (PMC9360403; doi:10.1038/s41598-022-17320-x)
Supplement: Supplementary file 1 — Supplementary Information. [file 41598_2022_17320_MOESM1_ESM.docx]

Impact on capsule formation for three different types of implant surface tomography

Hyeon Jun Jeon, MD, PhD^1^, MyeongJae Kang, MD^1^, Joon Seok Lee, MD, PhD^1^, Jieun Kang^2^, Eun A Kim, PhD^3^, Hee Kyung Jin, PhD^4^, Jae-sung Bae, PhD^5^, Jung Dug Yang, MD, PhD^1^*

^1^ Department of Plastic and Reconstructive Surgery, Kyungpook National University School of Medicine, Daegu, Korea

^2^ Cell and Matrix Research Institute, Kyungpook National University School of Medicine, Daegu, Korea

^3^ Exosome Convergence Research Center, Kyungpook National University School of Medicine, Daegu, Korea

^4^ Department of Laboratory Animal Medicine, College of Veterinary Medicine, Kyungpook National University, Daegu, Korea

^5^ Department of Physiology, Cell and Matrix Research Institute, Kyungpook National University School of Medicine, Daegu, Korea

**Corresponding author:**

Jung Dug Yang, MD, PhD.

Department of Plastic and Reconstructive Surgery

Kyungpook National University School of Medicine

130 Dongdeok-ro, Jung-gu, Daegu 700-421, Korea

[lambyang@knu.ac.kr](mailto:lambyang@knu.ac.kr)

**Supplementary information**

We would like to discuss the western blot results a little more. The original images with full-length blots were supplemented for the results that were not mentioned because they were cropped a lot in figure 5. of the manuscript.

In addition to this, the blots did not cut prior to hybridisation with antibodies. (A) on the supplementary figure is taken with chemluminesence and (B) shows the blot that is merged with the marker by illuminating the light (colormetric) to take the marker.


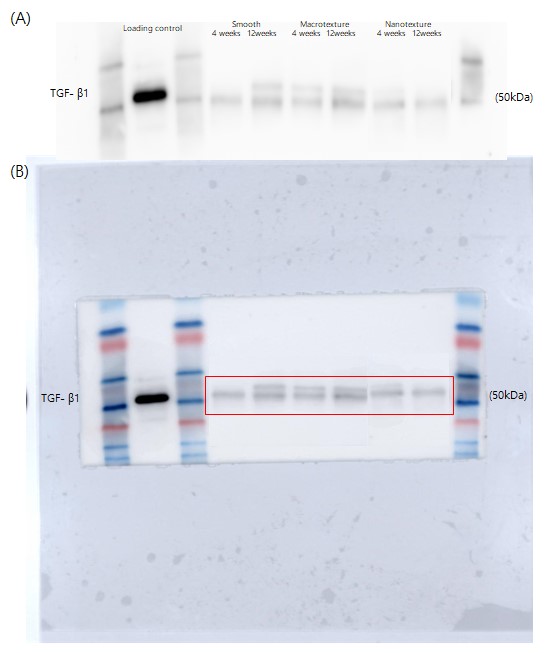


**Supplementary Figure 1.** Western blot analysis of TGF-β1 in the three different types of silicone implants


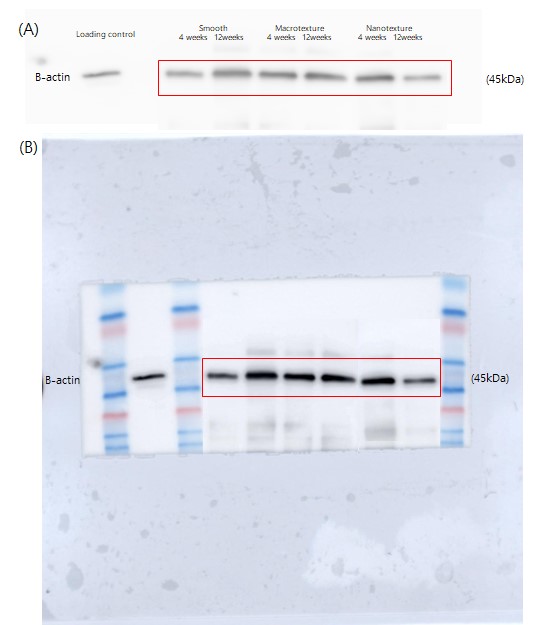


**Supplementary Figure 2.** Western blot analysis of β-actin in the three different types of silicone implants
